# Insights into the genetic history of Green-legged Partridgelike fowl: mtDNA and genome-wide SNP analysis

**DOI:** 10.1111/age.12046

**Published:** 2013-04-24

**Authors:** M Siwek, D Wragg, A Sławińska, M Malek, O Hanotte, JM Mwacharo

**Affiliations:** * Department of Animal Biotechnology, University of Technology and Life SciencesMazowiecka 28, 85-084, Bydgoszcz, Poland; † Centre for Genetics and Genomics, School of Biology, University Park, The University of NottinghamNottingham, NG7 2RD, UK; ‡ FAO/IAEA Agriculture and Biotechnology Laboratory, International Atomic Energy AgencyWagramerstrasse 5, 1400, Vienna, Austria

**Keywords:** autosomal markers, European chicken, *Gallus gallus*, mtDNA control region, Z chromosome markers.

## Abstract

The Green-legged Partridgelike (GP) fowl, an old native Polish breed, is characterised by reseda green-coloured shanks rather than yellow, white, slate or black commonly observed across most domestic breeds of chicken. Here, we investigate the origin, genetic relationships and structure of the GP fowl using mtDNA D-loop sequencing and genome-wide SNP analysis. Genome-wide association analysis between breeds enables us to verify the genetic control of the reseda green shank phenotype, a defining trait for the breed. Two mtDNA D-loop haplogroups and three autosomal genetic backgrounds are revealed. Significant associations of SNPs on chromosomes GGA24 and GGAZ indicate that the reseda green leg phenotype is associated with recessive alleles linked to the *W* and *Id* loci. Our results provide new insights into the genetic history of European chicken, indicating an admixd origin of East European traditional breeds of chicken on the continent, as supported by the presence of the reseda green phenotype and the knowledge that the GP fowl as a breed was developed before the advent of commercial stocks.

## Introduction

The Red junglefowl *Gallus gallus* is widely recognised as the main matriarchal ancestor of all domestic chicken (West & Zhou 1988), although it has recently been shown that other *Gallus* species also have contributed to the genetic make-up of some modern day domestic stocks (Nishibori *et al*. 2005; Eriksson *et al*. 2008). Initial evidence indicated that all domestic chicken originated from mainland South-East Asia (present day Thailand) from a single wild subspecies *G. g. gallus* (Fumihito *et al*. 1994, 1996). However, the recent observation of nine distinct mtDNA D-loop haplogroups in Eurasian domestic chicken has provided some support for multiple domestications across South-West and South-East Asia (Liu *et al*. 2006; Miao *et al*. 2012).

The genetic diversity of European native chicken at the mtDNA level remains poorly studied. A recent study of Hungarian chicken (Revay *et al*. 2010) revealed two mtDNA D-loop lineages, with the authors suggesting that these could have been derived from the Indian subcontinent and South-East Asia/China respectively. In a similar study, Dana *et al*. (2010) showed that East Asian chickens contributed to the development of Dutch traditional and Western commercial breeds of chicken.

The majority of European breeds have yellow-coloured shanks, whilst in others, they are typically white, bluish grey, slate or black. The Green-legged Partridgelike (GP; *Zielononóżka kuropatwiana*) fowl is an old native Polish breed of chicken with reseda green feet/shanks (Fig. S1) and grey partridgelike *Perdix perdix* plumage. The origin and development of the breed remain unclear. Most information about the breed originates from anecdotal reports (see: http://www.fao.org/ag/againfo/programmes/documents/genetics/story/story40.html). Polish chickens that phenotypically resembled GP fowls were described in 1866 by Pietruski and later on by Graff (1887). Other reports indicate that GP-like chicken were first introduced to Poland from Italy much earlier, possibly in the 16th century (Witkowski *et al*. 2009). Whatever the case, the name Green-legged Partridgelike was used for the first time in 1879 to denote the breed in the journal *Poultry Breeder* by Obfidowicz (Witkowski *et al*. 2009). In 1894, the breed was presented for the first time at a country exhibition in Lvov/Lviv, Ukraine. Other information from grey literature indicates that GP chickens were first bred in Poland in 1904. The breed was defined as a formal strain of the indigenous fowl of Poland in 1923, and from amongst several colour varieties, the Partridgelike feathers, reseda green legs/shanks and beaks were then chosen as the defining breed standards. In 1932, a herd book and nucleus breeding flock were established. The breed was registered in 1988 with the International Registry of Poultry Genetic Stocks (Somes 1988). Although for many years, the breed was widespread across Poland, by 1973, its population size had declined to almost 2% of its original size, and by 1988, only 624 birds were known to exist (Witkowski *et al*. 2009). Currently, the breed is maintained in two separate conservation flocks, each with about 600 hens and 70 cocks that are managed by the University of Life Sciences in Lublin and the Polish Institute of Animal Genetics and Breeding respectively. Fancy breeders also maintain a small number of unregistered flocks. The breed requires minimal veterinary health care, tolerates very low temperatures, is an excellent forager and is reputed to lay eggs low in cholesterol (Krawczyk *et al*. 2005; Krawczyk & Szefer 2012). In recent times, it has been used to study the genetic background of general immune responsiveness to KLH, LTA and LPS antigens (Siwek *et al*. 2010; Sławińska *et al*. 2011; Siwek *et al*. 2012).

The two most common skin pigments in birds are melanins and carotenoids. Melanins are divided into eumelanin, which is the main pigment in black or blue feathers, eyes, skin and connective tissue, and pheomelanin, which is deposited mainly in reddish brown and buff-coloured feathers. Carotenoids and xanthophylls are responsible for the yellow colouration in skin, fat and egg yolk (Smyth 1990). A wide spectrum of leg/shank colour polymorphisms (black, white, yellow, green, etc.) can be observed in domestic and wild chicken. In wild *Gallus* species, shank colours include shades of plumbeous brown to bluish grey (Red junglefowl, *G. gallus*), shades of yellow to salmon red (Grey junglefowl, *G. sonneratii*), brownish yellow (Ceylon junglefowl, *G. lafayetii*) and white to pink (Green junglefowl, *G. varius*) (Delacour 1977). In domestic chicken, yellow, white, grey, slate and/or black shanks are commonly observed. Some breeds such as Ayam Cemani, Black H’Mong, Korean Ogol, Kadaknath and Silkie have black skin and shanks. There are also various shades of either blue or green or both depending on the interaction of genes affecting the pigmentation of epidermis and dermis (Smyth 1990).

The major loci controlling skin colour phenotypes and their respective genotypes have previously been described (Bateson 1902; Dunn 1925; Dunn & Jull 1927; Hutt 1949). The genetic control of skin phenotypes relies on the cumulative and interactive effects of several major pigmenting loci. These include alleles for dominant white (wild type) and recessive yellow skin at the autosomal *W* locus, sex-linked dermal melanin (wild type) recessive to its inhibitor at the *Id* locus, epidermal melanin at the *E* locus, fibromelanosis at the *Fm* locus and several unidentified modifiers that enhance or restrict eumelanin (Smyth 1990). The green shank colour has previously been shown to result from the interaction of the *w*/*w* and *id*^*+*^*/id*^*+*^ alleles in the Cornell random-bred White Leghorn population (McGibbon 1974, 1979).

Eriksson *et al*. (2008) identified the *BCDO2* (also known as *BCO2*) as the gene underlying the yellow skin colour. The gene spans a region between 6.26 and 6.29 Mb in size on chromosome 24 (GGA24). Beta-carotene dioxygenase-2, the enzyme encoded by the *BCDO2* gene, acts by diffusing colourful carotenoids into colourless apocarotenoids, and this changes the skin colour from yellow to white.

The biology and genetic control of dermal hyperpigmentation (black skin/shanks) in the Silkie also have been studied (Dorshorst *et al*. 2010). Significant associations were mapped for two loci: dermal melanin (*Id*) to the Z chromosome (GGAZ) at position 72.3 Mb and fibromelanosis (*Fm*) to chromosome 20 (GGA20) at position 10.3–13.1 Mb. A further study by Dorshorst *et al*. (2011) identified the causal mutation of *Fm* as an inverted duplication and junction of two genomic intervals – one of which contains the *EDN3* gene, which plays a role in melanoblast proliferation.

In this study, we investigate the genetic relationship and structure of the GP fowl using mitochondrial DNA D-loop and full genome-wide SNP scan analysis. We reveal that the breed has an admixed genetic background from two maternal and three autosomal genetic contributions. We confirm that one of its defining traits, the reseda green shank phenotype, is associated with recessive alleles at both the *W* and *Id* loci. In the light of the known history of the breed, our results support the fact that admixed populations such as the GP fowl were likely present in Europe prior to the development of modern commercial breeds.

## Materials and methods

### Sample collection and DNA extraction

Eighty-four DNA samples [The Roslin Institute, *n* = 43; AVIANDIV (http://aviandiv.tzv.fal.de/), *n* = 8; private breeders, *n* = 33] representing 35 traditional European breeds of chicken were used for genotyping (Table 1). Junglefowl samples were obtained from various sources. *G. g. murghi* DNA samples were from Lehr Brisbin’s flock in Alabama, USA. Blood spotted on FTA® classic cards (Whatman) from two individuals each of *G. g. gallus*, *G. lafayettii*, *G. varius* and *G. sonneratii* came from a private breeder (Fazanterie de Rooie Hoeve, The Netherlands). For these samples, DNA was extracted using University of Nottingham’s in-house protocols. SNP data for a single individual each of *G. g. gallus*, *G. g. jabouillie*, *G. lafayettii* and *G. sonneratii* were provided by the Smurfit Institute, Dublin. For GP chicken, DNA was extracted from whole blood using the QuickExtract™ DNA extraction kit (Epicentre Biotechnologies) following the manufacturer’s protocol. Phenotypes for the loci of interest were inferred from literature and breed standards (Delacour 1977; Roberts 1997; Smyth 1990; Scrivener 2006, 2009), whereas those that were not fixed in any breed were recorded as unknown.

**Table 1:** Details of the breeds that were used for genotyping using the 60K SNP chip.

		Sample source and size			Genotypes^2^			
Breed	Shank colour^1^	RI	PB	AD	*W*^+^	*Id*	*Fm*	QC fail (IBS)
Domestic chicken								
Appenzellor	Blue	2			*W*^+^	*id*^+^/*id*^*+*^	*fm*^+^/*fm*^+^	1
Araucana	Willow to olive or slate	6	1		*w*/*w*	*id*^+^/*id*^*+*^	*fm*^+^/*fm*^+^	0
Brahma	Orange yellow or yellow	1			*w*/*w*	*Id*	*fm*^+^/*fm*^+^	0
Buff Orpington	White	1			*W*^+^	*Id*	*fm*^+^/*fm*^+^	0
Cochin	Yellow	1			*w*/*w*	*Id*	*fm*^+^/*fm*^+^	0
Cream Legbar	Yellow		2		*w*/*w*	*Id*	*fm*^+^/*fm*^+^	0
Crevecoeur	Black or slate blue		3		*W*^+^	*id*^+^/*id*^+^	*fm*^+^/*fm*^+^	0
Croad Langshan	Bluish black	1			*W*^+^	*id*^+^/*id*^+^	*fm*^+^/*fm*^+^	0
Derbyshire Redcap	Slate blue	1			*W*^+^	*id*^+^/*id*^+^	*fm*^+^/*fm*^+^	0
Dorking	White	1	2		*W*^+^	*Id*	*fm*^+^/*fm*^+^	0
Green-legged Partridgelike	Reseda green		5		*w*/*w*	*id*^+^/*id*^+^	*fm*^+^/*fm*^+^	0
Hamburgh	Lead blue	2			*W*^+^	*id*^+^/*id*^+^	*fm*^+^/*fm*^+^	1
Indian Game	Rich orange or yellow	1			*w*/*w*	*Id*	*fm*^+^/*fm*^+^	0
Ixworth	White	1			*W*^+^	*Id*	*fm*^+^/*fm*^+^	0
Leghorn	Yellow or orange	1			*w*/*w*	*Id*	*fm*^+^/*fm*^+^	0
Lincolnshire Buff	White	1			*W*^+^	*Id*	*fm*^+^/*fm*^+^	0
Malay	Rich yellow	1			*w*/*w*	*Id*	*fm*^+^/*fm*^+^	0
Marans	White	4	4	2	*W*^+^	*Id*	*fm*^+^/*fm*^+^	0
Marsh Daisy	Pale willow green	1			*w/w*	*id*^+^/*id*^+^	*fm*^+^/*fm*^+^	0
Modern Langshan	Transparent White	1			*W*^+^	*Id*	*fm*^+^/*fm*^+^	0
Norfolk Grey	Black	1			*W*^+^	*id*^+^/*id*^+^	*fm*^+^/*fm*^+^	0
Old English Pheasant Fowl	Slate blue	1			*W*^+^	*id*^+^/*id*^+^	*fm*^+^/*fm*^+^	0
Polish	White or bluish white	2			*W*^+^	*Id*	*fm*^+^/*fm*^+^	0
Rhode Island Red	Yellow or red horn	1			*w*/*w*	*Id*	*fm*^+^/*fm*^+^	0
Scots Dumpy	Black or slate on black	1			*W*^+^	*id*^+^/*id*^+^	*fm*^+^/*fm*^+^	0
Scots Grey	White or white with black spots	1			*W*^+^	*Id*	*fm*^+^/*fm*^+^	0
Silkie	Lead	4			*W*^+^	*id*^+^/*id*^+^	*Fm*	0
Spanish	Pale slate blue	1			*W*^+^	*id*^+^/*id*^+^	*fm*^+^/*fm*^+^	0
Sultan	White or pale blue		1		*W*^+^	*Id*	*fm*^+^/*fm*^+^	0
Sussex	White	4			*W*^+^	*Id*	*fm*^+^/*fm*^+^	0
Totenko	Olive green		7		*w/w*	*id*^+^/*id*^+^	*fm*^+^/*fm*^+^	5
Villafranquina	Slate blue			2	*W*^+^	*id*^+^/*id*^+^	*fm*^+^/*fm*^+^	0
Welsummer	Yellow		5		*w*/*w*	*Id*	*fm*^+^/*fm*^+^	0
White Star	Yellow		2		*w*/*w*	*Id*	*fm*^+^/*fm*^+^	0
Yurlov	Yellow or black			4	?	?	*fm*^+^/*fm*^+^	0
Junglefowls			PB	SI				
*Gallus gallus gallus*	Plumbeous brown to bluish grey		2	1	*W*^+^	?		0
*Gallus gallus murghi*	Plumbeous brown to bluish grey		3		*W*^+^	?		2
*Gallus gallus jabouillei*	Plumbeous brown to bluish grey			1	*W*^+^	?		0
*Gallus lafayetti*	Brownish yellow		2	1	w/w	?		3
*Gallus varius*	White to pink		2		?	?		1
*Gallus sonneratii*	Shades of yellow to salmon red		2	1	w/w	?		2

Sources: RI, Roslin Institute; PB, Private Breeder; AD, AvianDiv; ILRI, International Livestock Research Institute; SI, Smurfit Institute); Genotype (?, unknown phenotype);

1 Delacour (1977), Roberts 1997, Smyth (1990), Scrivener (2006, 2009).

2 Allele combinations inferred from shank colour and Smyth (1990).

### mtDNA studies

For mtDNA D-loop analysis, DNA was extracted from whole blood from 31 GP birds from a conservation flock of the experimental farm of the University of Life Sciences, Lublin, Poland. This flock has been maintained as a closed population since 1960 and can be regarded as a true and pure representative of GP fowls. DNA was extracted using the DNeasy® Blood and Tissue kit (Qiagen). The first 800 bp of the mtDNA D-loop region was amplified via PCR and sequenced using primer pair L16750 (5′-AGGACTACGGCTTGAAAAGC-3′) and CR1b (5′-CCATACACGCAAACCGTCTC-3′) following Mwacharo *et al*. (2011).

## Data analysis

### mtDNA D-loop sequences

For each sample, two fragments were generated. These were edited manually using bioedit 7.0 (Hall 1999) and then aligned against a reference sequence (GenBank accession number NC_007235; Nishibori *et al*. 2005) using clustalx 2.0.12 (Thompson *et al*. 1997). Subsequent analyses were restricted to the first 397 bp of the D-loop spanning the first hypervariable region (HVR I). dnasp 4.0 (Rozas *et al*. 2003) was used to define the haplotypes. arlequin v3.5.1.2 (Excoffier & Lischer 2010) was used to estimate haplotype and nucleotide diversities and the mean number of pairwise differences and to infer historical population dynamics from mismatch distribution patterns. Departure of the observed mismatch distribution from the simulated model of demographic expansion was tested with chi-squared test of goodness of fit and Harpending’s (1994) raggedness index ‘*r*’ following 10 000 coalescent simulations. These two tests were augmented with the Fu (1997) ‘*F*_S_’ and Tajima (1996) ‘*D*’ coalescent-based estimators of neutrality and whose significance were also tested with 10 000 simulations. To assess the number of haplogroups in the GP fowl, a median-joining (MJ) network (Bandelt *et al*. 1999) was constructed using network 4.5 (fluxus-engineering.com). To portray the affinity of GP to other Eurasian chicken, the construction of the MJ network included 40 haplotypes downloaded from GenBank (Table S1). Nine were from Liu *et al*. (2006), 11 from Revay *et al*. (2010) and 20 from Dana *et al*. (2010). For this purpose, the central and most common haplotypes for the different clades/haplogroups observed in these three studies were selected to infer the maternal origins of GP within the geographic range of the wild ancestor, the Red junglefowl and their possible shared maternal ancestries with other European traditional breeds.

### SNP analysis of genetic relationships, structure and association mapping

All samples were genotyped by a private company (DNA Landmarks, Inc.). SNP genotyping was performed using the 60K SNP Illumina iSelect™ chicken microarray (Groenen *et al*. 2011; http://www.illumina.com/). Further details may be found in Wragg *et al*. 2012. structure v2.3.3 (Pritchard *et al*. 2000) was used to evaluate the genetic structure of the study populations using a subset of the data that included a single individual selected at random from each breed and 40 000 randomly selected SNPs. A model assuming admixture between populations with unlinked loci was used for the analysis with a burn-in of 50 000 and Markov chain Monte Carlo repetitions of 100 000 assuming 1 ≤ *K* ≤ 5 genetic clusters.

The approach used for association mapping has been described in detail in Wragg *et al*. 2012. Individuals with unknown phenotypes were excluded. SNP genotyping data were pruned using the genabel package (Aulchenko *et al*. 2007) for r (R Core Team 2009). Initially, the mean and standard deviation of identity-by-state (IBS) scores were calculated across all samples using the ibs function. The check.marker function was then used to prune the data set using the following attributes: maf = 0.05, call = 0.9, per.id = 0.9, p.lev = 0, ibs.mrk = ‘ALL’, ibs.threshold = ibscut, ibs.exclue = ‘lower’. The ibscut score was set to 0.7849959, a value that corresponds to the mean plus three times the standard deviation of IBS scores. All birds that failed this threshold are presented in Table 1 under the column heading QC fail (IBS). The qtscore function of genabel was used to perform tests of allelic associations, with reported *P* values corrected to one degree of freedom – equivalent to the Armitage test. Genotype frequencies were calculated across all samples, including those that initially failed QC, using the summary.snp.data function for each significant SNP mapping to previously published regions for *W* (Eriksson *et al*. 2008), *Id* and *Fm* (Dorshorst *et al*. 2010) loci. Frequencies were calculated for all birds and then independently for Silkie, junglefowl and GP.

## Results

### mtDNA D-loop analysis

**Figure 1:**
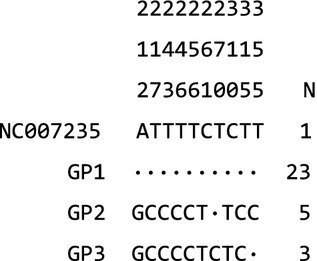
Sequence variation of three haplotypes derived from 31 Green-legged Partridgelike chickens observed in the mtDNA D-loop region. Mutations are scored relative to the reference sequence (GenBank accession number NC007235; Nishibori *et al*. 2005). Dots (·) denote nucleotide identity with the reference sequence. Numbers in the first three lines read vertically represent the positions of the variable sites with respect to the reference sequence. The number of individuals sharing the same haplotypes is indicated in the right column by ‘N’. These haplotypes have been deposited with the NCBI under accession numbers KC560148–KC560150.

**Figure 2:**
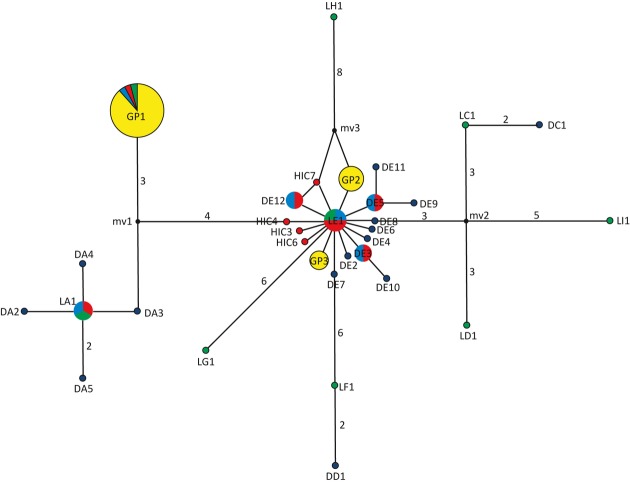
Median-Joining network of the three haplotypes of Green-legged Partridgelike fowl (GP1, GP2, GP3) and 40 haplotypes downloaded from the GenBank (Supplementary Table 1) and representing Eurasian (haplotypes starting with letter ‘L’; Liu *et al*. 2006), Hungarian (haplotypes starting with ‘HIC’ Revay et al. 2010) and Dutch fancy and commercial breeds (haplotypes starting with letter ‘D’; Dana *et al*. 2010) of chicken. Circled areas are proportional to haplotype frequencies. Median vectors are represented by ‘mv’. Unless shown by using numbers, all interlinked haplotypes are separated by a single mutation. Key to colour codes: Blue = Haplotypes from Dutch fancy and commercial breeds, Green = Haplotypes of Eurasian chicken, red = Haplotypes of Hungarian chicken, Yellow = Haplotypes of Green-legged Partridgelike chicken. Circles in which more than two of these colours occur means that the haplotypes are shared between the respective groups of chicken represented by each colour. With the exception of haplotype GP1 which is shared between Eurasian, Dutch fancy and Hungarian indigenous chickens, for haplotypes shared between Eurasia, Dutch and Hungarian indigenous chickens, haplotype names (those starting with letter ‘L’ from Liu *et al*. (2006) are adopted while for haplotypes shared between Dutch fancy breeds and Hungarian indigenous chickens, haplotype names (those starting with letter ‘D’) from Dana *et al*. (2010) are used.

Sequences spanning the first 397 bp of the mtDNA D-loop and which included the HVR I were analysed. From 31 sequences, three haplotypes defined by 10 polymorphic sites were generated (Fig. 1). For this study, haplotypes observed in GP are abbreviated ‘GP’ followed by a numeral, for example GP1 (Figs 1 and 2). Haplotype GP1 (with 23 sequences) was the most common, whereas GP2 (with five sequences) and GP3 (with three sequences) were the least frequently observed. Haplotype and nucleotide diversities for the GP fowl were 0.362 ± 0.210 and 0.0080 ± 0.0021 respectively, whereas the average number of nucleotide differences between sequences was 3.626 ± 1.888.

The mismatch distribution pattern for the GP deviated significantly (calculated SSD = 0.217, *P* = 0.024) from a unimodal distribution pattern typical of populations that have experienced recent or past demographic expansions. The GP’s distribution was characterised by three distinct peaks at 0, 2 and 9 mismatches respectively (Fig. S2). The failure to reject the null hypothesis was further supported by a non-significant Harpendings raggedness index (*r* = 0.642, calculated *P* = 0.294). Both Fu’s *F*_S_ and Tajima’s *D* statistics were positive (*F*_S_ = 7.229; *D* = 1.410), indicating a decline in population size in the recent past and/or balancing selection.

Together with the 40 haplotypes downloaded from GenBank, the MJ network revealed a grouping of the three GP haplotypes (GP1, GP2 and GP3) into two haplogroups (Fig. 2). The first haplogroup is made up of haplotype GP1 that is associated with clade B of Liu *et al*. (2006) (Table S2a). This haplotype is identical to haplotypes B1 (Liu *et al*. 2006), HIC11 (Revay *et al*. 2010) and B1 (Dana *et al*. 2010) observed in Eurasian, Hungarian and Dutch traditional and commercial breeds respectively (Table S2b). The haplogroup involving these haplotypes also was observed in commercial broilers and traditional chickens from North-West Europe by Muchadeyi *et al*. (2008) and in a single individual from Ethiopia by Mwacharo *et al*. (2011). Liu *et al*. (2006) proposed Yunnan province in China and/or adjacent areas as the possible centre of origin of this haplogroup. The second haplogroup is made up of haplotypes GP2 and GP3 (Fig. 2). These two are closely related to haplotype E1 from clade E of Liu *et al*. (2006), haplotypes HIC1–HIC9 observed in Hungarian chicken (Revay *et al*. 2010) and haplotypes E1–E12 observed in Dutch traditional and commercial breeds (Dana *et al*. 2010) (Table S2(a) and S2(b)). Haplotypes from this haplogroup predominate amongst African village chicken from Zimbabwe, Malawi, Sudan (Muchadeyi *et al*. 2008), Nigeria (Adebambo *et al*. 2010) and Kenya, Uganda, Ethiopia and Sudan (Mwacharo *et al*. 2011). Liu *et al*. (2006) observed this haplogroup in Europe, the Middle East and India and suggested that it could have its roots in the Indian subcontinent. Only two (B and E) of the nine (A to I) clades observed in Eurasian chicken by Liu *et al*. (2006) are found in the GP. These results suggest that, like other European chicken from Hungary and the Netherlands as well as commercial breeds, the GP has two maternal genetic backgrounds, one derived likely from the Indian subcontinent and the other possibly from southern China or northern South-East Asia.

### Genetic structure and association mapping

**Figure 3:**
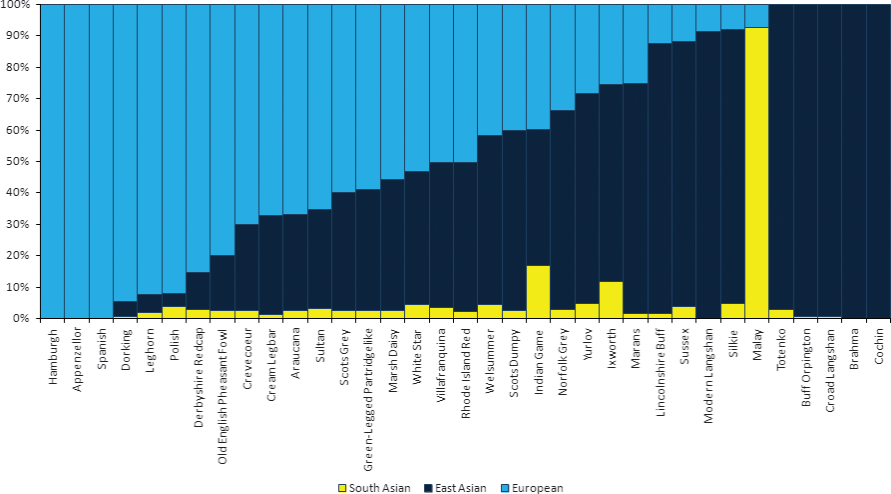
Genetic structure of the nuclear genome (autosomal and Z chromosome) for 35 breeds revealed from the analysis of 60K SNP microarray.

We partitioned genetic variation amongst the study breeds using the parametric genetic admixture algorithm implemented in structure 2.3.3 (Pritchard *et al*. 2000). Between one and five clusters (*K*) were tested assuming admixture between populations and linkage equilibrium between loci. A graphic representation of the estimated membership coefficient for each individual to each cluster at 1 ≤ *K* ≤ 5 is shown in Fig. 3. An estimate of the posterior probability of the data (Ln[P(D)]) indicated that *K* = 3 was the most optimal. With the exception of Hamburgh, Appenzellor, Spanish, Brahma and Cochin, all the other breeds appear to be admixed among the three clusters with the proportion of each cluster varying across breeds. We interpret these three clusters to represent European, East Asian and South Asian genetic backgrounds consistent with known breed histories (Scrivener 2006, 2009).

For each locus (*W*, *Id*, *Fm*), binomial association tests were performed on the pruned data set. After pruning, 29 birds with yellow shanks (*w*/*w*) and 50 with white shanks (*W*^*+*^) were retained for analysis. For the inhibitor of dermal melanin phenotype, two tests were performed as the phenotypes were uncertain based on inference from breed standards. In the first test, the phenotypes were assigned as per Table 1, resulting in 39 birds exhibiting the presence of dermal melanin (*id*^*+*^/*id*^*+*^) and 40 exhibiting its absence (*Id*). In the second test, only the Silkie birds and the GP were assigned the *id*^*+*^/*id*^*+*^ phenotype, whilst all the other birds were assigned the *Id* phenotype, resulting in nine birds exhibiting the presence of dermal melanin (*id*^*+*^/*id*^*+*^) and 74 exhibiting its absence (*Id*). Finally, dermal hyperpigmentation (*Fm*) is only present in the Silkie resulting in four birds with the *Fm* phenotype and 79 of the wild type (*fm*^+^/*fm*^+^).

Results of the top five significant outcomes from each binomial test are presented in Table 2. These are in agreement with previously published mapping data for the *W* and *Fm* loci (Eriksson *et al*. 2008; Dorshorst *et al*. 2010). In the table, those not highlighted are considered false positives for the purpose of this study. They are likely the result of the limited number of samples involved in each test. As already indicated, the *W* and *Fm* results are consistent with previous findings, although the mapping of the *Id* locus appears inconclusive. By considering only the Silkie and GP birds to possess *id*^*+*^/*id*^*+*^ and all the other birds to possess *Id*, the results agree with those of Dorshorst *et al*. (2010). However, applying the allelic interactions that are known to result in specific shank colours (Table 3) to the breed standards of each bird used for the test fails to map any significant associations to *Id*.

**Table 2:** Significant results following association tests for the loci *W, Id* and *Fm* using the 60K SNP chip.

Locus	Chromosome	Position	*P*-value	
W		24	6279104	4.72e–07
		24	6273988	4.95e–07
		24	6261633	1.97e–06
		12	11022567	3.31e–05
		1	19149987	6.98e–05
Id	Only GP and Silkie *id*^+^/*id*^+^	Z	72985598	2.11e–07
		2	138132039	1.05e–05
		3	41040834	3.00e–05
		12	7186919	3.22e–05
		Z	33105782	3.53e–05
	Id phenotypes as recorded in Table 1	9	12500113	6.99e–05
		13	5917154	8.39e–05
		1	158528977	2.28e–04
		13	5963541	2.84e–04
		5	54486865	4.25e–04
*Fm*		20	10990821	1.02e–06
		1	56353716	1.48e–06
		28	2986479	1.58e–06
		2	68439874	2.15e–06
		2	72080734	4.80e–06

Note. Chromosomal positions based on *galGal3* genome assembly. Results not highlighted are considered false positives for the purpose of this study and are likely the result of the limited number of samples involved in each test.

**Table 3:** Interactions of *W* and *Id* on shank colour.

Shank colour	*Id*	*W*
White	*Id*	*W*^*+*^
Yellow	*Id*	*w*/*w*
Green (willow)	*id*^*+*^/*id*^*+*^	*w*/*w*
Slate/Blue/Black	*id*^*+*^/*id*^*+*^	*W*^*+*^

Adapted from Smyth (1990).

**Figure 4:**
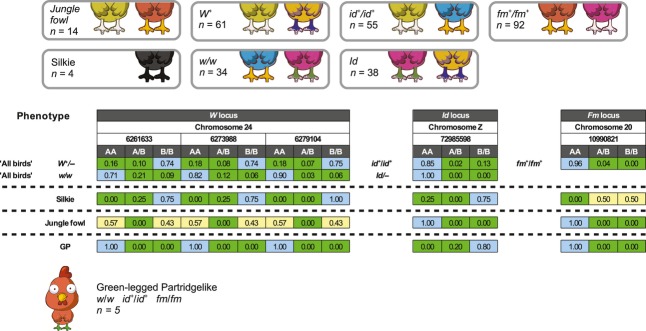
Genotype frequencies at *W*, *Id* and *Fm* loci-mapped SNPs controlling shank pigmentation.

After association testing, genotype frequencies for significant SNPs, identified in the previously mapped regions for each locus, were estimated (Fig. 4). Genotype frequencies were calculated as per the phenotypes recorded in Table 1 for all birds excluding the GP, junglefowl and the Silkie. The same alleles linked to yellow skin phenotype at the *W* locus were found to be present in the GP (Fig. 4), supporting that the locus contributes to the green leg/shank phenotype. As the junglefowl group included both white- and yellow-skinned species, a similar number of homozygotes were observed for each allele at the same locus. The genotype frequencies at the *Id* locus mapped SNP were less conclusive across all birds. However, a similar allele frequency at the SNP in the region previously mapped for the *Id* locus is observed in Silkie and GP. Finally, the SNP frequencies mapped for the *Fm* locus confirmed the allele as absent in the GP and in 96% of non-Silkie breeds, whilst one allele was observed in both heterozygous and homozygous forms in the Silkie, supporting a dominant mode of inheritance. None of the alleles for *Id* or *Fm* were observed in any of the junglefowl samples.

## Discussion

The GP chicken is an indigenous Polish breed, which at one point was threatened with extinction but currently is maintained in two independent conservation schemes (Witkowski *et al*. 2009). In this study, we investigate the origin of the breed and describe its genetic structure using the maternally inherited mtDNA D-loop and genome-wide SNP analysis (Groenen *et al*. 2011; Wragg *et al*. 2012). Using the latter, we demonstrate the likely genetic control of the reseda green-coloured shank phenotype, a standard trait for the breed. Our study reveals two distinct mtDNA D-loop haplogroups and three autosomal genetic clusters, indicating that the GP fowls, together with most European traditional breeds, have mixed maternal and possibly paternal ancestries. Due to its admixed nature, the expectation was that the breed would display relatively high genetic diversity. However, its haplotype and nucleotide diversities are lower than those observed in free-range scavenging village chicken from Asia (Liu *et al*. 2006; Silva *et al*. 2008) and Africa (Muchadeyi *et al*. 2008; Mwacharo *et al*. 2011). The contraction in population size (bottleneck) experienced by the breed in the 1970s, supported by positive Tajima’s *D* and Fu’s *F*_S_ statistics obtained in this study, might be responsible for the low levels of maternal diversity in the GP. The subsequent development and multiplication of the breed, from a small founder population with a narrow genetic base and the long-term maintenance of the breed as a closed flock, are also possible contributing factors.

We observed at least two mtDNA D-loop haplogroups (B and E) and three autosomal genetic clusters based on the 60K SNP chip analysis in the GP breed. The two mtDNA D-loop haplogroups are associated with similar haplogroups observed in other traditional breeds from Europe and Asia (Fig. 2). Similarly, the three autosomal genetic clusters are also present in other European traditional and fancy breeds of chicken included in the current study (Fig. 3). These results support a mixed maternal and paternal ancestry for the European breeds in general.

Moreover, our results suggest a distinct origin and history of the two haplogroups and the three autosomal genetic clusters observed in GP and European traditional breeds (Figs 2 and 3; Tables S2a and S2b). Indeed, the two mtDNA D-loop haplogroups are related to geographically distinct Asian mtDNA haplogroups, primarily deriving from Yunnan Province in China and/or adjacent regions (haplogroup B (GP1)) and South Asia [haplogroup E (GP2 and GP3)] (Liu *et al*. 2006). On the other hand, the three autosomal genetic clusters indicate the GP fowl and most European traditional breeds analysed here to be genetically admixed (Fig. 3). The presence of several different genetic backgrounds in the GP fowl and most European traditional breeds raises interesting questions on how, when and where such admixture could have taken place.

Recent cross-breeding with commercial breeds may have contributed to the admixture. Both commercial broiler and egg layers share the same mtDNA haplogroups B and E with the GP and European traditional breeds (Fig. 2; Dana *et al*. 2010). However, we believe this to be unlikely for the GP fowl. Indeed, there is no historical record indicating that recent commercial introgression might have occurred in the GP fowl, although it might have remained undocumented. Also, such introgression does not explain the presence of the reseda green shank phenotype in the breed. Furthermore, the reseda green shank phenotype was described prior to the development of commercial breeds (Pietruski 1866; Graff 1887).

Alternatively, the genome admixture present in the GP might have originated prior to the standardisation of the breed or during its formation. Our association analysis confirms that an interaction between the recessive yellow skin allele and the wild recessive allele at the dermal melanin locus present in the Silkie is responsible for the green shank phenotype. The failure to map a significant association at the *Id* locus using all breeds based on expected allelic combinations raises an interesting question: might there be multiple alleles for the *Id* locus as was also noted by Smyth (1990). The alternatives are that the phenotypes of the birds used do not meet breed standards or that none of the SNPs on the panel are in strong linkage disequilibrium with the locus in the other breeds analysed.

Eriksson *et al*. (2008) revealed that the allele responsible for the yellow skin phenotype is likely to have introgressed into domestic chicken from the Grey junglefowl *G. sonneratii*, which is endemic on the Indian subcontinent. The origin of the non-wild-type allele at the *Id* locus remains uncertain. All the junglefowl tested shared the same alleles at the SNP that was mapped for the *Id* locus in the Silkie and GP, suggesting that *Id* is possibly the result of a *de novo* mutation in domestic stocks.

The demonstration here that the reseda green shank phenotype in the GP fowl arises from the interaction of recessive alleles at the *W*^*+*^ and *Id* loci further supports at least two genetic backgrounds in the breed. These may have already been present in the ancestral Asian domestic populations or could have arisen following admixture on the European continent of chickens from different origins and/or introductions.

Literature suggests the geographic origin of the GP fowl to be the Mediterranean region, specifically Italy, from where it diffused to Poland in the 16th century (Witkowski *et al*. 2009). This seems to suggest that its hybrid origin may have occurred in Italy. In this respect, it is worth mentioning that the Sicilian Buttercup, which originally came from Sicily, has willow green shanks and feet (Roberts 1997; Scrivener 2006). The presence of green shanks in the GP fowl might have resulted from cross-breeding with white leg chicken carrying the sex-linked dermal melanin (*id*^+^/*id*^+^) alleles. Such chicken might have been, for example, Silkie-type birds known to have been present across Europe since at least the 16th century (Corti & Corti 2009).

Finally, in contrast to chicken from Africa and Asia, the diversity of indigenous European chicken is found today in fancy or traditional breeds rather than in village chicken populations. Often with small effective population sizes, these fancy breeds are exposed to various threats (FAO 2009) and are at risk of extinction (FAO 2007). There is a need to ensure that they are sustainably conserved. Such an action will help to achieve two major goals: 1) provide model organisms for academic and scientific research aimed, for example, at understanding the inheritance pattern and interaction pathways of genes underlying admixed phenotypes and 2) conserve the legacy of the intellectual ability, knowledge and efforts of past breeders in creating such unique phenotypes.

## Supporting information

Figure S1 Example of reseda green and yellow shank phenotypes in chicken. Picture courtesy of Ewa Gorzycka.

Figure S2 Mismatch distribution pattern for the mtDNA Dloop sequence haplotypes generated from 31 Green-legged Partridgelike chickens.

Table S1 Accession numbers for the 40 haplotypes that were downloaded from the GenBank and included in the study.

Table S2 a. Clustering patterns between the three haplotypes observed in Green-legged Partridgelike chickens and those downloaded from the GenBank as observed in the MJ Network; b. Clustering patterns for the three haplotypes observed in Green-legged Partridgelike chickens and those downloaded from the GenBank on the MJ Network.
