# Supplementary material for: Insights into the genetic history of Green-legged Partridgelike fowl: mtDNA and genome-wide SNP analysis
Source: Anim Genet. 2013 Apr 24;44(5):522–32. doi: 10.1111/age.12046 (PMC3793231; doi:10.1111/age.12046)
Supplement: Figure S1 — Example of reseda green and yellow shank phenotypes in chicken. Picture courtesy of Ewa Gorzycka. [file age0044-0522-sd1.pdf]

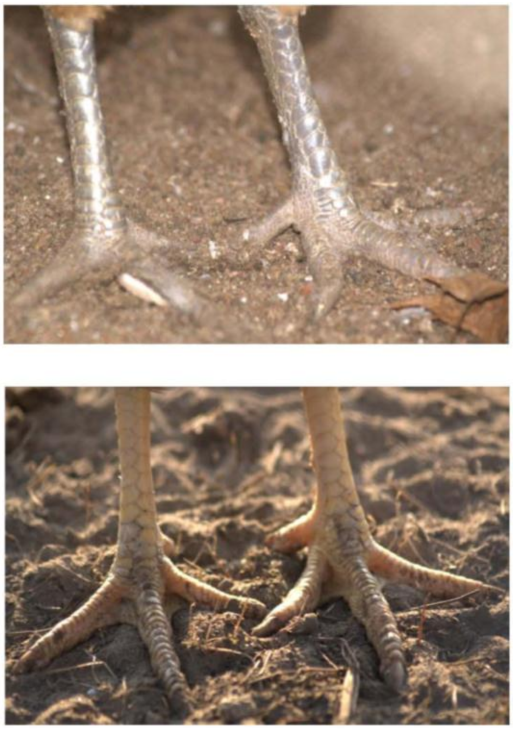

a) Reseda-green shanks

b) Yellow shanks

Figure S1. Example of reseda green and yellow shank phenotypes in chicken. Picture courtesy of Ewa Gorzycka.
