# Supplementary material for: Insights into the genetic history of Green-legged Partridgelike fowl: mtDNA and genome-wide SNP analysis
Source: Anim Genet. 2013 Apr 24;44(5):522–32. doi: 10.1111/age.12046 (PMC3793231; doi:10.1111/age.12046)
Supplement: Figure S2 — Mismatch distribution pattern for the mtDNA Dloop sequence haplotypes generated from 31 Green-legged Partridgelike chickens. [file age0044-0522-sd2.pdf]

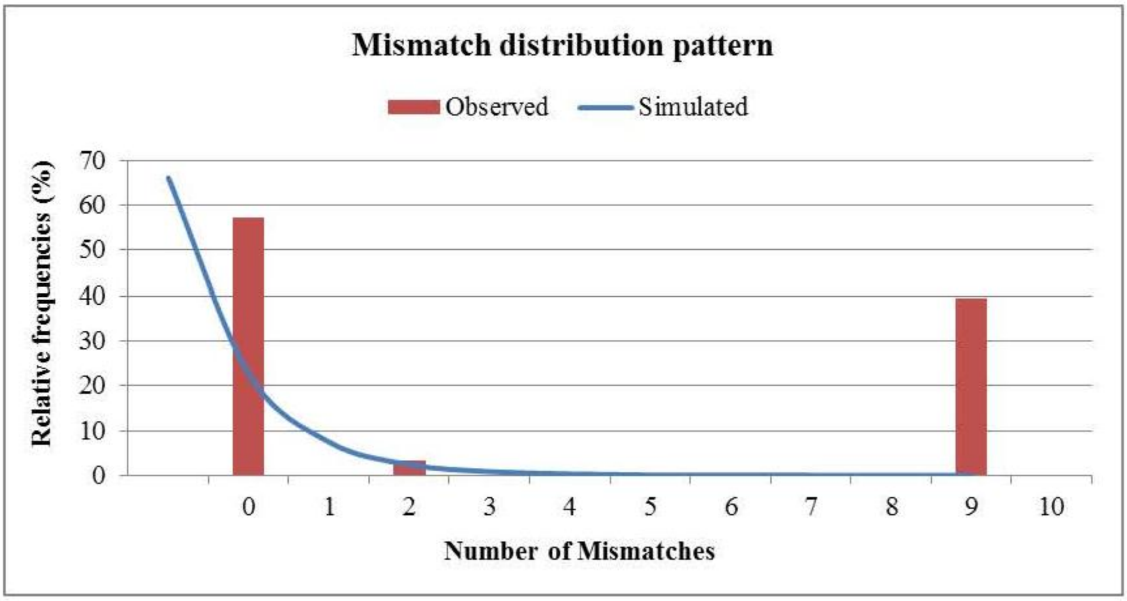

Figure S2. Mismatch distribution pattern for the mtDNA D-loop sequence haplotypes generated from 31 Green-legged Partridgelike chickens.
