# Supplementary material for: Insights into the genetic history of Green-legged Partridgelike fowl: mtDNA and genome-wide SNP analysis
Source: Anim Genet. 2013 Apr 24;44(5):522–32. doi: 10.1111/age.12046 (PMC3793231; doi:10.1111/age.12046)
Supplement: Table S1 — Accession numbers for the 40 haplotypes that were downloaded from the GenBank and included in the study. [file age0044-0522-sd3.pdf]

Table S1. Accession numbers for the 40 haplotypes that were downloaded from the GenBank and included in the study

Accession Number	Haplotype	Country	Source/author
HM015602	A1	The Netherlands	Dana *et al* (2010)
HM015603	A2	"	"
HM015604	A3	"	"
HM015605	A4	"	"
HM015606	A5	"	"
HM015607	B1	"	"
HM015608	C1	"	"
HM015609	D1	"	"
HM015610	E1	"	"
HM015611	E2	"	"
HM015612	E3	"	"
HM015613	E4	"	"
HM015614	E5	"	"
HM015615	E6	"	"
HM015616	E7	"	"
HM015617	E8	"	"
HM015618	E9	"	"
HM015619	E10	"	"
HM015620	E11	"	"
HM015621	E12	"	"
GQ258689	HIC1	Hungary	Revay *et al* (2010)
GQ258690	HIC2	"	"
GQ258691	HIC3	"	"
GQ258692	HIC4	"	"
GQ258693	HIC5	"	"
GQ258694	HIC6	"	"
GQ258695	HIC7	"	"
GQ258696	HIC8	"	"
GQ258697	HIC9	"	"
GQ258698	HIC10	"	"
GQ258699	HIC11	"	"
AB114069	A1	Europe and Asia	Liu *et al* (2006)
AB007744	B1	"	"
AB114070	C1	"	"
AY588636	D1	"	"
AB114076	E1	"	"
AF512285	F1	"	"
AF512288	G1	"	"
D82904	H1	"	"
AB009434	I1	"	"
