# Supplementary material for: Insights into the genetic history of Green-legged Partridgelike fowl: mtDNA and genome-wide SNP analysis
Source: Anim Genet. 2013 Apr 24;44(5):522–32. doi: 10.1111/age.12046 (PMC3793231; doi:10.1111/age.12046)
Supplement: Table S2 — a. Clustering patterns between the three haplotypes observed in Green-legged Partridgelike chickens and those downloaded from the GenBank as observed in the MJ Network; b. Clustering patterns for the three haplotypes observed in Green-legged Partridgelike chickens and those downloaded from the GenBank on the MJ Network. [file age0044-0522-sd4.pdf]

Table S2a. Clustering patterns between the three haplotypes observed in Green-legged Partridgelike chickens and those downloaded from the GenBank as observed in the MJ Network

Haplotypes observed in this study	Associated haplotypes from other studies		
	**Liu *et al*. 2006**	**Dana *et al*. 2010**	**Revay *et al*. 2010**
GP1	- Haplotype B1 from Clade B (observed in Yunnan China)<br>- Suggested center of origin: Yunnan China and/or surrounding areas	Haplotype B1	Haplotype HIC11
GP2 and GP3	- Haplotype E1 from Clade E (observed in Europe, Middle East and India)<br>- Possible postulated center of origin: Indian subcontinent	Haplotypes E1, E2, E3, E4, E5, E6, E7, E8, E9, E10, E11, E12	Haplotypes HIC1, HIC2, HIC3, HIC4, HIC5, HIC6, HIC7, HIC8, HIC9

Table S2b. Clustering patterns for the three haplotypes observed in Green-legged Partridgelike chickens and those downloaded from the GenBank on the MJ Network

	**Haplotypes included in the MJ Network**			
Haplotype name in MJ Network	Liu *et al*. 2006	Revay *et al*. 2010	Dana *et al*. 2010	This study
GP1	B1	HIC11	B1	GP1
GP2				GP2
GP3				GP3
LA1	A1	HIC10	A1	
DA2			A2	
DA3			A3	
DA4			A4	
DA5			A5	
DC1			C1	
DD1			D1	
LE1	E1	HIC1, HIC9	E1	
DE2			E2	
DE3		HIC5	E3	
DE4			E4	
DE5		HIC2	E5	
DE6			E6	
DE7			E7	
DE8			E8	
DE9			E9	
DE10			E10	
DE11			E11	
DE12		HIC8	E12	
HIC3		HIC3		
HIC4		HIC4		
HIC6		HIC6		
HIC7		HIC7		
LC1	C1			
LD1	D1			
LF1	F1			
LG1	G1			
LH1	H1			
LI1	I1			
